# Trace Voltammetric Determination of Lead at a Recycled Battery Carbon Rod Electrode

**DOI:** 10.3390/s19040770

**Published:** 2019-02-13

**Authors:** Kevin Honeychurch

**Affiliations:** Faculty of Applied Sciences, University of the West of England, Frenchay Campus, Coldharbour Lane, Bristol, BS16 1QY, UK; Kevin.honeychurch@uwe.ac.uk

**Keywords:** lead, cyclic voltammetry, stripping voltammetry, recycled, carbon, environmentally friendly

## Abstract

Carbon rod electrodes (CREs) were obtained from recycled zinc–carbon batteries and were used without further modification for the measurement of trace concentrations of lead (Pb). The electrochemical behavior of Pb at these electrodes in a variety of supporting electrolytes was investigated by cyclic voltammetry. The anodic peaks obtained on the reverse scans were indicative of Pb being deposited as a thin layer on the electrode surface. The greatest signal–to–noise ratios were obtained in organic acids compared to mineral acids, and acetic acid was selected as the supporting electrolyte for further studies. Conditions were optimized, and it was possible to determine trace concentrations of Pb by differential pulse anodic stripping voltammetry. A supporting electrolyte of 4% v/v acetic acid, with a deposition potential of −1.5 V (vs. SCE) and a deposition time of 1100 s, was found to be optimum. A linear range of 2.8 µg/L to 110 µg/L was obtained, with an associated detection limit (3σ) of 2.8 µg/L. A mean recovery of 95.6% (CV=3.9%) was obtained for a tap water sample fortified with 21.3 µg/L.

## 1. Introduction

The toxicity of Pb is widely understood [1]. As the knowledge in the field of toxicology has developed, it has become increasingly apparent that there is no safe level of Pb exposure. Consequently, a number of different measures have been undertaken to try and lower our exposure, such as the removal of Pb from petrol, paint, glazes and pigments. As a result, food and drinking water now represent the principle sources for Pb exposure [2]. The present limit for Pb in drinking water supplies across the European Union (EU) is 10 µg/L [3], which is in line with the World Health Organization (WHO) health limit [4]. The EU recognizes Pb as one of the substances known to cause direct health impacts, and as a result has proposed to lower the drinking water limit to 5 µg/L [5]. However, a number of reports from both the EU [6] and regions outside, such as the USA [7], Australia [8] and Pakistan, [9] have highlighted Pb drinking water levels that exceed this level. 

Obviously, there is a pressing need for methods capable of determining trace concentrations of Pb in both potable and environmental water samples. A range of different analytical methods have been utilized, including inductively coupled plasma and atomic adsorption spectroscopy. However, these are comparatively expensive techniques, requiring well-equipped laboratories with highly trained staff for their implementation. Alternative techniques such as anodic stripping voltammetry have also been employed, but have suffered from the use of relatively large amounts of Hg required for their application. However, a number of Hg-free devices have been reported, made possible by either the in situ or ex situ deposition of other less toxic metal films, such as Bi [10], Sb [11] or, more recently, Cu [12] and Sn [13]. Nevertheless, some disadvantages of the application of metal film-based electrodes have been highlighted [14]. The application of a metal film can result in the narrowing of the usable potential range, and therefore interfere with the determination of metals such as Hg and Cu. The relatively large concentrations of the film forming metal ion in solution can also affect the speciation of the target analytes. As with most thin-film techniques, peak splitting can also be observed. Alternatively, as shown in Table 1, studies have also tended to focus on the development of different films and membranes to enhance the analytical and performance characteristics of the developed working electrode. By using such an approach, it has been possible to gain low detection limits and selectively determine metal ions in complex samples. For example, Deshmukh et al. [15] have recently shown the possibility of utilizing a stainless steel EDTA–PANI/SWCNT nanocomposite electrode for the simultaneous trace determination of Cu, Pb and Hg. However, this requires a well-equipped laboratory and experienced personnel for their development and fabrication. Their complexity could also lead to issues with subsequent mass production.

An alternative approach that can demonstrate similar performance characteristics (Table 1) is the possibility of utilizing the direct deposition, and subsequent stripping, of the target ion at unmodified carbon electrode surfaces. This technique has suffered in the past from issues of multiple stripping peaks being formed due to the heterogeneous nature of the electrode surface [16]. However, studies have shown the possibility of direct accumulation and measurement of Pb [17], Cu [18] and Zn [19] at carbon electrodes. 

Another possible source of carbon electrodes is via the recycling of Zn–carbon batteries. These batteries are comprised of a Zn outer case (can) serving as the anode. Contained inside is a moist powdered mixture of carbon, ammonium chloride, and manganese (IV) oxide as the cathode. Also contained within is a carbon rod, which collects the current from the interaction of the Zn and the manganese (IV) oxide. The Zn–carbon battery represents a large percentage of the market and is generally recycled using a number of different approaches [20]. However, little has been reported on the direct reuse of some of its internal components.

This present investigation focuses on exploring the voltammetric behavior of Pb at carbon rod electrode (CRE) extracted from a Zn–carbon battery. The application of this carbon rod as a working electrode offers economic advantages, is readily available, and is a green, environmental alternative to other more expensive and commonly employed electrodes. The proposed method described here was based on the direct deposition and stripping of Pb from the carbon electrode surface obtained from a spent recycled 1.5 V Zn–carbon battery. A 4% v/v acetic acid solution, similar in concentration to vinegar, was utilized as the supporting electrolyte; materials that were recycled or exhibited low toxicity and environmental impact. In the first part of this investigation, the cyclic voltammetric behavior of Pb at the carbon rod electrode (CRE) was investigated. The second section focuses on optimizing the conditions required for the trace determination of Pb by differential pulse anodic stripping voltammetry. In the third and final section, the possibility of determining trace concentrations of Pb in a potable tap water samples was investigated.

## 2. Materials and Methods

### 2.1. Chemical and Reagents

All chemicals were supplied from Fisher (Loughborough, UK), unless otherwise stated. Lead stock solutions were prepared by dissolving the appropriate mass of Pb(NO_3_)_2_ (Sigma, Poole, Dorset, UK) in deionized water (Purite Select Analyst 80 System, Purite Oxon, UK). Working standards were then prepared by dilution of the primary stock solution with deionized water. Supporting electrolyte solutions for cyclic voltammetric and differential pulse anodic stripping voltammetry (DPASV) studies were prepared by dilution of the salt or acid under investigation. A 4% v/v acetic acid solution was made by diluting 2 mL of acetic acid to give a total volume of 50 mL with deionized water. Tap water samples were obtained from the potable water supply in the laboratory. The tap was run for 2 min prior to sample collection. These were then adjusted to be 4% v/v acetic acid.

### 2.2. Apparatus

Cyclic voltammetry and DPASV were performed using a Pstat10 potentiostat interfaced to a PC for data acquisition via the General Purpose Electrochemical System Software Package (GPES) version 3.4 (Autolab, The Netherlands). Six mm diameter carbon rod working electrodes were extracted from a spent, recycled 1.5 V zinc–carbon electrode (R14L, Panasonic, Kadoma, Japan) obtained from the university’s recycling center. 

### 2.3. Scanning Ectron Mcroscopy (SEM) and Eergy-Dspersive X-ray Sectroscopy (EDX)

SEM was undertaken using a Philips XL30 ESEM system. EDX examinations of the electrode surface were undertaken using an Oxford Instruments Link ISIS 3.2 EDX system. 

### 2.4. Fabrication of the Carbon Rod Electrode

A suitable 1.5 V Zn–carbon battery was cut open and the carbon rod removed. This was then washed with deionized water to remove lose adhering material. The carbon rod was then sonicated in separate 200 mL portions of deionized water until they remained clear. The resulting CRE was then removed and dried with tissue. The 6 mm diameter end was isolated with tape (RS components, Corby, UK), connected to the potentiostat and employed as the working electrode in a three electrode system, consisting of a saturated calomel reference electrode (SCE) and a carbon rod counter electrode. 

### 2.5. Voltammetric Procedures

Cyclic voltammograms were initially recorded in plain solutions of the supporting electrolyte under investigation, and then in the same solution containing 0.1 mM of Pb^2+^ purged with oxygen-free nitrogen. The cyclic voltammetric conditions were as follows: initial potential, 0.0 V; scan rate, 50 mV/s; and switching potential, −1.5 V. DPASV was undertaken using a deposition time of 1100 s at −1.5 V (vs. SCE) while stirring the solution via a magnetic stirrer bar (HI-180F Compact Magnetic Mini Stirrer, Hanna Instruments, Bedfordshire, UK). Following deposition, the solution was left in quiescence for 15 s. The voltammogram was then recorded from −1.5 V to 0.0 V using a step height of 2.4 mV, pulse repetition time of 0.2 s, pulse height of 50 mV and pulse width of 50 ms.

## 3. Results and Discussion

### 3.1. EDX Examination of the CRE 

**Figure 1:**
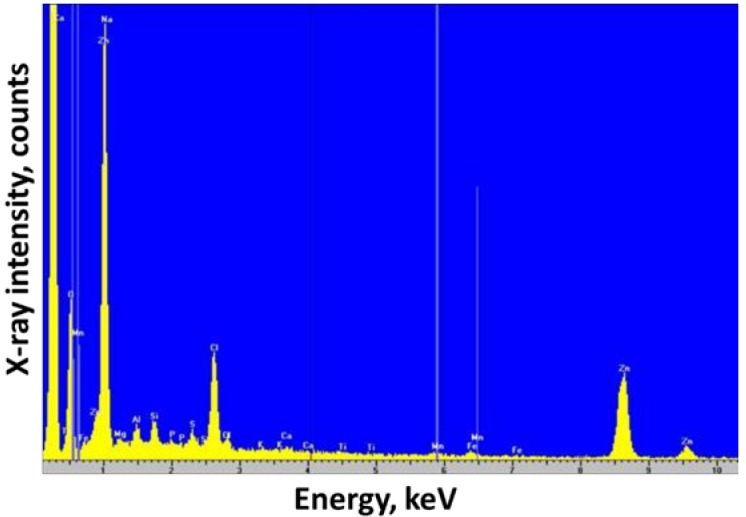
Energy-dispersive X-ray spectroscopy of the carbon rod surface.

Figure 1 shows a typical EDX spectrum obtained for the CRE surface. EDX investigations were undertaken to explore the possible presence of metals sorbed on the carbon structure. In subsequent applications, these could interfere with the determination of Pb or other metals. Both Mn and Zn were recorded, presumed to result from the cathode and anode of the Zn–carbon battery, along with notable signals for calcium, in agreement with that previously reported [30]. However, this signal was more likely the result of the Kα line for carbon. A strong peak was recorded at *ca.* 1 keV, which could be associated with the Kα line for sodium. However, it was more likely the result of the Lα line for zinc. No evidence was recorded for the presence of other metals, allowing the application of the CRE for the determination of Pb. 

### 3.2. Cyclic Voltammetric Behavior of Lead at Bare CREs

**Figure 2:**
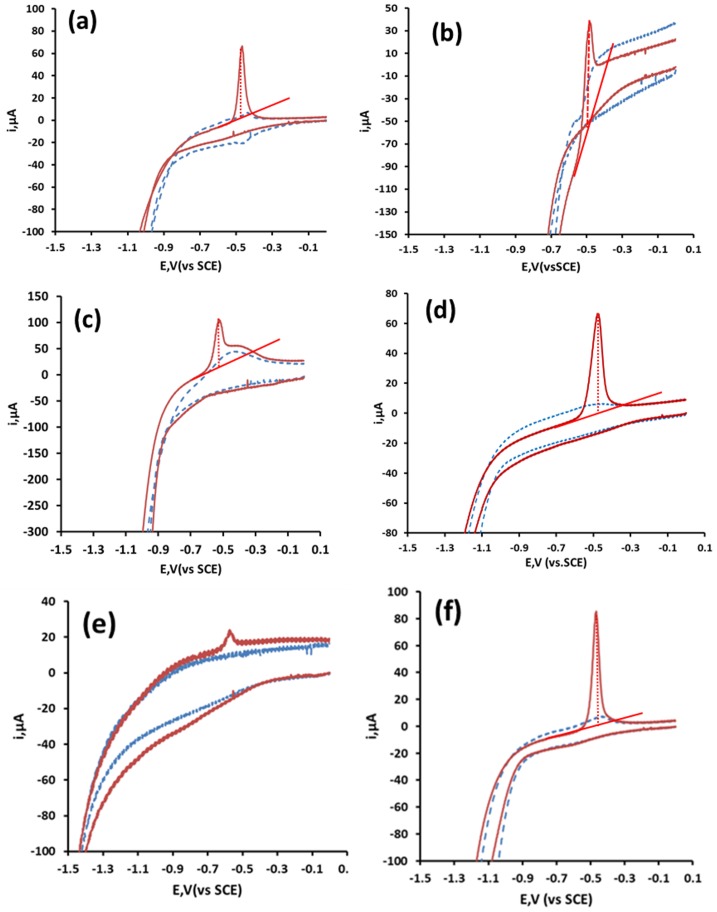
Effect of supporting electrolyte on the cyclic voltammetric behavior of Pb. (**a**) Ortho-phosphoric acid, (**b**) nitric acid, (**c**) hydrochloric acid, (**d**) acetic acid, (**e**) potassium chloride and (**f**) malonic acid. Blue dashed line in the absence, and red solid line in the presence of 116 µM Pb.

Figure 2 shows typical cyclic voltammograms obtained in a selection of supporting electrolytes for 116 µM Pb solution at the CRE. Generally, the resulting voltammograms exhibited one anodic peak on the return positive scan (Ep ca. −0.5 V), which resulted from the stripping of Pb metal deposited at the electrode surface. Little evidence of any cathodic processes was recorded, but was presumed to be masked by the simultaneous reduction of the background supporting electrolyte. The formation of a metal film at the electrode surface was necessary for the accumulation step in anodic stripping voltammetry and offered the possibility of utilizing these CREs for the determination of Pb by anodic stripping voltammetry. 

The different behaviors observed for the supporting electrolytes that were investigated were notable. Those undertaken in organic acids generally showed improved voltammetric behavior over those undertaken in mineral acids and salts. Hysteresis was observable when utilizing ortho-phosphoric acid, and more notably for nitric acid as supporting electrolytes (Figure 2a,b, a phenomenon often seen during the deposition of metal ions on foreign electrode material surfaces. This resulted from differences in the nucleation over potentials required for deposition of Pb^2+^ cations onto carbon on the initial scan, compared to that required for deposition to the Pb film formed during the return scan [31]. The Pb stripping peak obtained with HCl as a supporting electrolyte (Figure 2c) suffered from interference by a broad peak present both in the presence and absence of Pb between −0.48 V and −0.20 V when employing HCl. Both these effects made baseline assignment difficult. Also notable is the poor response in neutral or basic supporting electrolytes. No anodic peaks were recorded when utilizing disodium phosphate as the supporting electrolyte (not shown), presumably due to the formation of insoluble lead phosphate. However, an anodic peak was recorded when using phosphoric acid (Figure 2a). Only a small stripping peak was found while utilizing the commonly employed electrolyte potassium chloride (Figure 2e). Unlike the results shown here, previous studies at carbon electrodes [32] have shown that the presence of chloride ions greatly improved the voltammetric behavior of Pb. Well-defined Pb stripping peaks were obtainable when using 1.0 M acetic acid (Figure 2d) and malonic acid (Figure 2f) as the supporting electrolytes. As acetic acid has been successfully employed in previous studies [19], this supporting electrolyte was explored in further investigations.

### 3.3. Effect of Supporting Electrolyte Concentration

**Figure 3:**
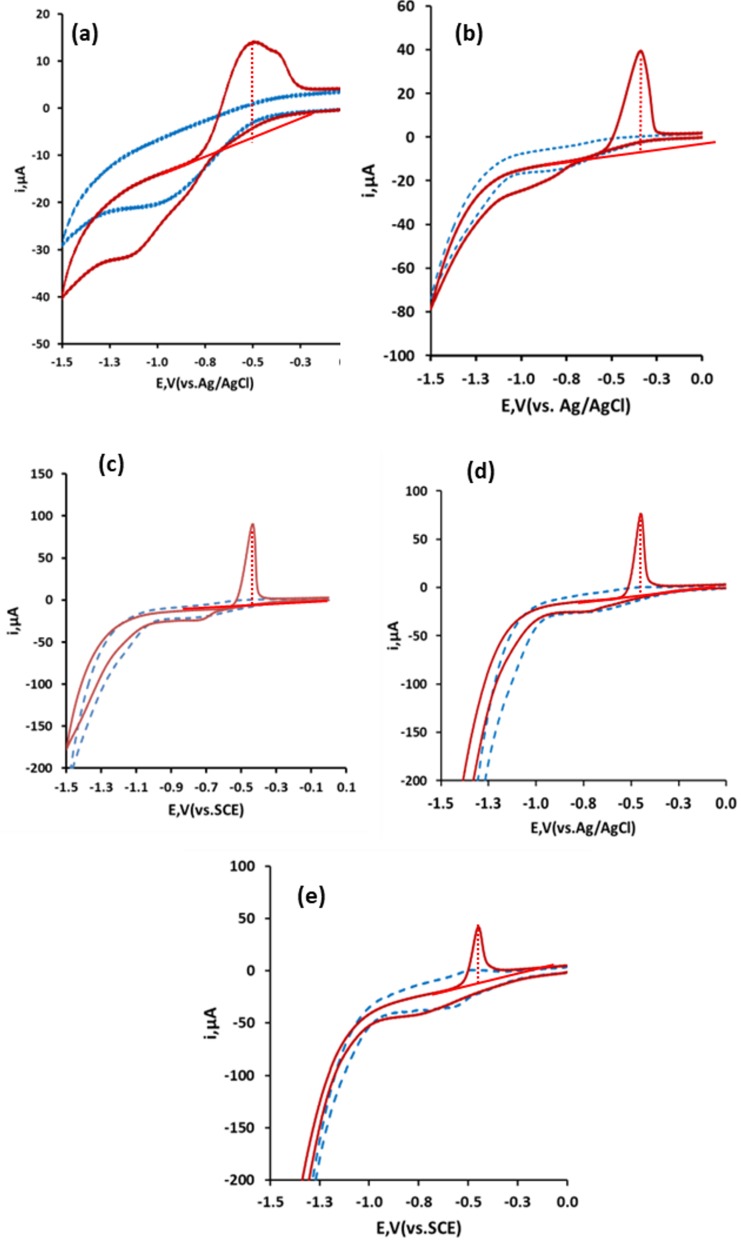
Effect of acetic acid supporting electrolyte concentration on the cyclic voltammetric behavior of 116 µM Pb: (**a**) 0.0 M, (**b**) 0.03 M, (**c**) 0.1 M, (**d**): 0.66 M and (**e**) 3.0 M.

The concentration and nature of the supporting electrolyte, having a number of effects, is known to be highly important [33]. The effect of varying the concentration of acetic acid on the resulting cyclic voltammetric behavior was investigated over the range 0.03 M to 3.0 M for a 116 µM Pb solution (Figure 3). Below an acetic acid concentration of 0.1 M, both the magnitude and shape of the Pb stripping peak were found to be inferior. The optimum voltammetric behavior was seen with acetic acid concentrations between 0.1 M and 1.0 M. One of the aims of this investigation was to develop an environmentally friendly analytical method. A 0.66 M acetic acid solution represented a 4% v/v concentration, similar to that of vinegar, and was hence used in further studies.

### 3.4. Differential Pulse Anodic Stripping Voltammetry

**Figure 4:**
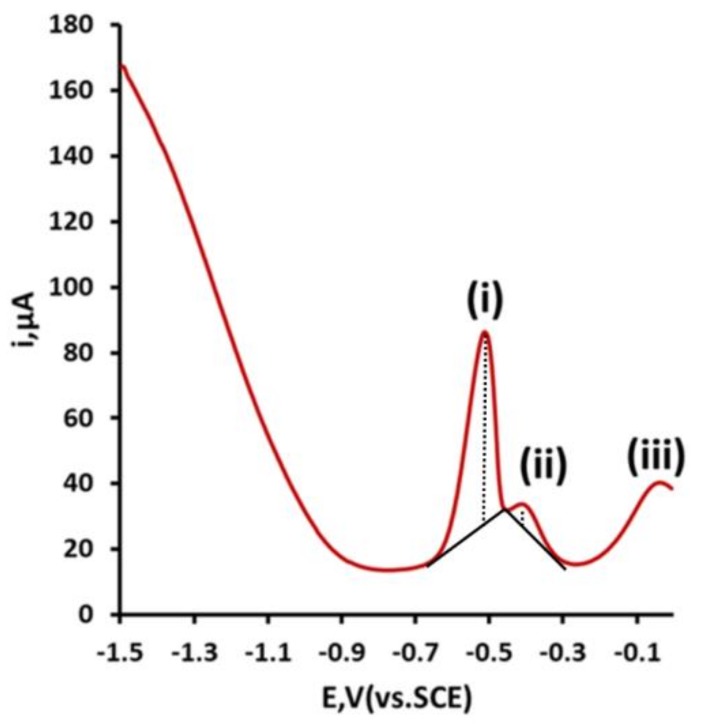
Differential pulse anodic stripping voltammetry of 100 µg/L Pb. voltammetric conditions: deposition potential, −1.5 V (vs. SCE); deposition time, 14 min.

In previous studies of carbon electrodes, it has been shown that multiple stripping peaks can be recorded for Pb [17] and other metals [16]. This arises from the heterogeneous nature of the electrode surface. As a result, metal ions were deposited on the electrode surface not as a continuous layer but as islands, mono, or multiple layers with differing affinities to the surface. This was consequently reflected in the stripping step, where these different affinities for the electrode surface required different applied potentials to be removed (stripped) from the surface. As can be seen in Figure 4, the CRE showed very similar behavior to that previously reported at other carbon electrodes [17]. Three stripping peaks, which have been denoted as (i), (ii) and (iii), were recorded, with Ep values of −0.57 V, −0.44 V and −0.05 V, respectively.

### 3.5. Effect of Accumulation Potential

**Figure 5:**
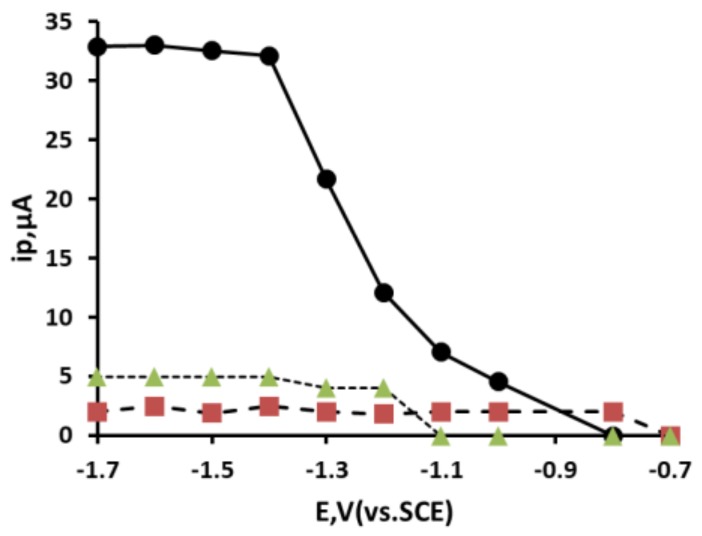
Effect of deposition potential. Peak (i) black circle, peak (ii) red square and peak (iii) green triangle.

The effect of accumulation potential was studied for a 0.1 mM Pb^2+^ solution over the range of −0.7 V to −1.7 V (vs. SCE) using an accumulation time of 45 s with stirring, followed by 15 s in quiescence (Figure 5). Under these conditions, the magnitude of the three stripping peaks, (i), (ii) and (iii), was found to increase as the accumulation potential was made more negative. Both the processes resulting in peaks (i) and (ii) required accumulation potentials more negative than −0.7 V (vs. SCE). However, peak (iii) required negative potentials beyond −1.2 V (vs. SCE). The peak current of peak (i) was found to give the largest magnitude forming a plateau between −1.3 V and−1.7 V (vs. SCE). Consequently, further studies were focused on this peak and an accumulation potential of −1.5 V (vs. SCE) was used in additional investigations.

### 3.6. Effect of Accumulation Time

**Figure 6:**
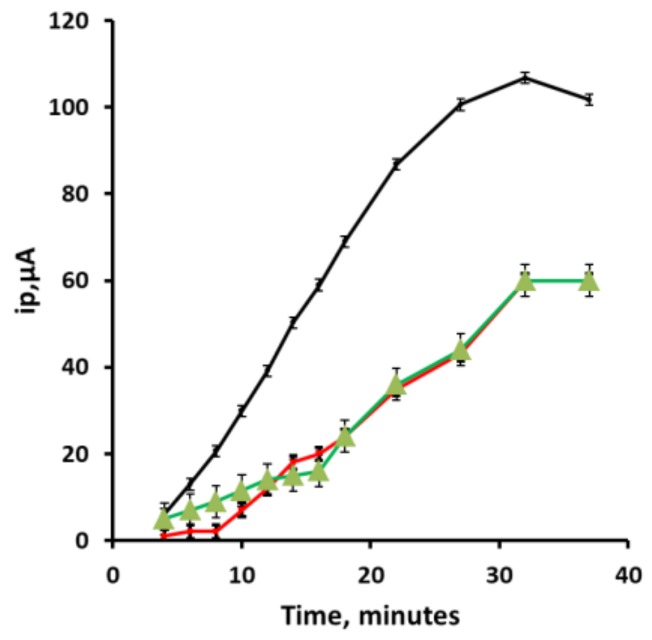
Effect of deposition time for a 100 µg/L Pb^2+^ solution in the optimized electrolyte. Error bars represent plus and minus a standard deviation.

Figure 6 shows the relationship for the peak current (ip) values of peaks (i), (ii) and (iii) for 4% v/v acetic acid solution containing 100 µg/L Pb. All three peaks were found to increase in magnitude with increasing time and to plateau after 30 min for a 100 µg/L Pb^2+^ solution. Peak currents for all three anodic Pb stripping peaks were found to increase linearly with time. Peak (i) exhibited a near linear relationship with time for up to 20 min (76.6 nA/s, R^2^ = 0.999), becoming independent of time beyond this point. 

### 3.7. Effect of Pb Concentration

**Figure 7:**
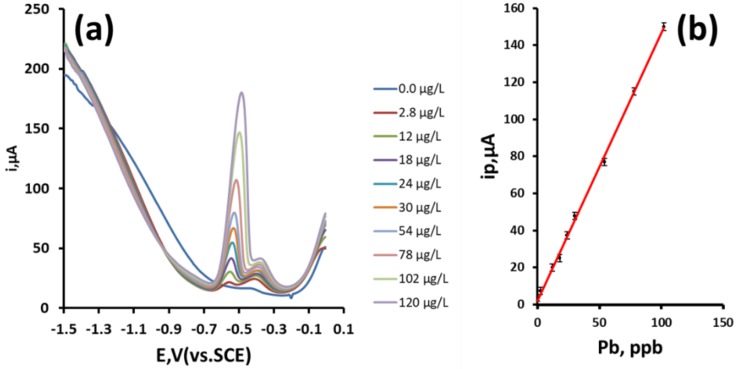
(**a**) DPASVs obtained for increasing Pb concentrations over the range 2.8 µg/L to 120 µg/L; (**b**) resulting calibration curve. Error bars represent plus and minus a standard deviation.

Figure 7 shows the DPASVs obtained in 4% acetic acid from 2.8 µg/L to 120 µg/L Pb. Utilizing peak (i) and an accumulation time of 1100 s with forced convection at an applied potential of −1.5 V (SCE), a linear relationship with Pb^2+^ concentration and ip was obtained from 2.8 µg/L to 110 µg/L (R^2^ = 0.999, 1.45 µA/ng/mL). Based on a signal–to–noise ratio of three (3σ), a theoretical detection limit of 2.8 µg/L was calculated. 

### 3.8. Analytical Application

The CREs were evaluated by carrying out Pb determinations on tap water before and after fortifying with Pb at a concentration of 21 µg/L, using the optimized DPASV parameters used previously. The concentration of Pb was determined by multiple standard addition. Table 2 shows the precision and recovery data obtained for a tap water sample. The results indicate that the proposed method could be applied to the determination of Pb in potable tap water samples.

## 4. Conclusions

The redox behavior of Pb has been investigated at carbon electrodes fabricated from a Zn–carbon 1.5 V recycled battery. A well-defined anodic peak could be obtained in several of the supporting electrolytes investigated. However, the optimum response was obtained in an electrolyte comprised of 4% v/v acetic acid. Utilizing DPASV with a deposition potential of −1.5 V for 1100 s, a linear range of 2.8 µg/L to 110 µg/L with an associated detection limit of 2.8 µg/L was obtained. Performance characteristics, which are comparable or better to that obtained at much more expensive electrodes, such as boron-doped diamond electrodes [34], can be obtained directly by either inductively coupled plasma atomic emission spectroscopy [35,36] or atomic absorption spectroscopy [37]. A mean recovery of 95.6% with an associated coefficient of variation of 3.9% was obtained for a potable tap water sample fortified with 21 µg/L Pb. In further investigations, the possible application CREs for the determination of other metals and drugs will be explored. Alternatively, the cheap, disposable properties of these CREs could be employed for the electrochemical cleaning of Pb-rich industrial wastewater.

## Figures and Tables

**Table 1:** Summary of the recent application of carbon working electrodes of the stripping voltammetric determination of metal ions.

Working Electrode Material	Linear Range	Detection Limit	Voltammetric Technique	Sample	Ref.
Stainless steel EDTA–PANI/SWCNT nanocomposite electrode	Pb(II) ca. 400 µg/L–1500 µg/L; Cu(II) ca. 120 µg/L–6000 µg/L and Hg(II) ca. 400 µg/L–20,000 µg/L	Cu(II), 5.1 µg/L; Pb(II) 342 µg/L and Hg(II) 136 µg/L	DPASV ^1^	/	[15]
Paper-based electrode using *Cladophora rupestris* cellulose coated with polyaniline (PANI)	0.2 mg/L–1.0 mg/L	Pb(II) 72 µg/L	LSASV ^2^	/	[21]
Carbon paste electrode modified with *Eichhornia crassipes* powder	Pb (II) and Cd (II) 10 µg/L 5000 μg/L for Cd(II) and Pb (II)	4.9 μg/L Cd(II), 2.1 μg/L Pb(II)	SWASV ^3^	Natural water samples	[22]
Multiwall carbon nanotube MWCNT/(H_2_bpabza) novel tetradentate carboxamide ligand modified electrode	Pb(II) 1.72 µg/L– 23.8 µg/L; Cd(II) 0.930 µg/L–12.3 µg/L	Pb(II) 0.56 µg/L, Cd(II) 0.10 µg/L	SWASV ^3^	Rice and tap water samples	[23]
Porous activated carbon-supported, palladium nanoparticles-modified glassy carbon electrode	Cd(II) 5.6 µg/L–56.2 µg/L Pb(II) 10.3 µg/L–104 µg/L Cu(II) 3.2 µg/L −32 µg/L	Cd(II) 2.3 µg/L, Pb(II) 1.90 µg/L, Cu(II) 0.97 µg/L	SWASV ^3^	/	[24]
Zeolitic imidazolate chitosan-modified glassy carbon electrode	Hg(II) 1.0 μM–80.0 μM, Cu(II), 64 µg/L–6.4 mg/L Pb(II) 207 µg/L−20.7 mg/L Cd(II) 112 μg/L−11.2 mg/L	Hg(II) 5.86 µg/L, Cu(II) 6.96 µg/L, Pb(II) 12.8 µg/L, Cd(II) 15.2 µg/L	DPASV ^1^	Lake water	[25]
Fully 3-D printed carbon nanofiber–graphite–polystyrene electrode	Zn(II) 12.7 μg/L−450 μg/L	Zn(II) 8.6 μg/L	DPASV ^1^	Tap water	[19]
Diamond/graphite nanoplatelet electrode	10 μg/L−250 μg/L	Zn(II) 1.72 μg/L, Cd(II) 0.47 μg/, Pb(II) 4.86 μg/L Cu(II), 0.45 μg/L	DPASV ^1^	/	[26]
Nanoporous bismuth-modified electrode	5.0 µg/L−40 µg/L	1.3 µg/L Cd(II), 1.5 µg/L Pb(II)	SWASV ^3^	Tap water	[27]
Single-walled carbon, nanohorns-modified, bismuth film screen-printed electrode	1.0 μg/L−60 μg/L	0.2 μg/L Cd(II), 0.4 μg/L Pb(II)	SWASV ^3^	Honey and milk samples	[28]
Porous graphitic carbon nitride nanosheets and oxidized multiwalled carbon nanotube-modified screen-printed carbon electrode	Hg(II) 4.8 µg/L− 93.0 µg/L, Pb(II) 0.35 µg/L−6.5 µg/L and 6.5 µg/L−110 µg/L, Cd(II) 4.25 µg/L−79.0 µg/L and 79.0 µg/L−251 µg/L, Zn(II) 4.2 µg/L−202 µg/L	Hg(II) 0.04 µg/L, Pb(II) 0.008 µg/L, Cd(II) 0.03 µg/L, Zn(II) 0.06 µg/L	DPASV ^1^	Vegetables (cabbage and capsicum) and food products (noodles)	[29]
Unmodified battery carbon rod electrode	2.8 µg/L–110 µg/L Pb(II)	2.8 µg/L Pb(II)	DPASV ^1^	Tap water	This work

^1^ Differential pulse anodic stripping voltammetry, ^2^ linear sweep anodic stripping voltammetry and ^3^ square wave anodic stripping voltammetry.

**Table 2:** Precision and recovery data for the DPASV determination of lead in tap water.

	Original Concentration, µg/L	Added, µg/L	Found, µg/L	% Recovery
1	ND	21.3	20.3	95.3
2	ND	21.3	19.3	90.6
3	ND	21.3	19.8	93.0
4	ND	21.3	21.5	101
5	ND	21.3	20.9	98.3

Mean recovery = 95.6%; coefficient of variation = 3.9%; ND = not detected.
